# Circadian Rhythms Disrupted by Light at Night and Mistimed Food Intake Alter Hormonal Rhythms and Metabolism

**DOI:** 10.3390/ijms24043392

**Published:** 2023-02-08

**Authors:** O. Hecmarie Meléndez-Fernández, Jennifer A. Liu, Randy J. Nelson

**Affiliations:** Department of Neuroscience, Rockefeller Neuroscience Institute, West Virginia University, Morgantown, WV 26506, USA

**Keywords:** circadian rhythms, hormonal rhythms, SCN, sleep, metabolism, light, melatonin, jet lag, cardiovascular

## Abstract

Availability of artificial light and light-emitting devices have altered human temporal life, allowing 24-hour healthcare, commerce and production, and expanding social life around the clock. However, physiology and behavior that evolved in the context of 24 h solar days are frequently perturbed by exposure to artificial light at night. This is particularly salient in the context of circadian rhythms, the result of endogenous biological clocks with a rhythm of ~24 h. Circadian rhythms govern the temporal features of physiology and behavior, and are set to precisely 24 h primarily by exposure to light during the solar day, though other factors, such as the timing of meals, can also affect circadian rhythms. Circadian rhythms are significantly affected by night shift work because of exposure to nocturnal light, electronic devices, and shifts in the timing of meals. Night shift workers are at increased risk for metabolic disorder, as well as several types of cancer. Others who are exposed to artificial light at night or late mealtimes also show disrupted circadian rhythms and increased metabolic and cardiac disorders. It is imperative to understand how disrupted circadian rhythms alter metabolic function to develop strategies to mitigate their negative effects. In this review, we provide an introduction to circadian rhythms, physiological regulation of homeostasis by the suprachiasmatic nucleus (SCN), and SCN-mediated hormones that display circadian rhythms, including melatonin and glucocorticoids. Next, we discuss circadian-gated physiological processes including sleep and food intake, followed by types of disrupted circadian rhythms and how modern lighting disrupts molecular clock rhythms. Lastly, we identify how disruptions to hormones and metabolism can increase susceptibility to metabolic syndrome and risk for cardiovascular diseases, and discuss various strategies to mitigate the harmful consequences associated with disrupted circadian rhythms on human health.

## 1. Introduction

For the past three to four billion years, life on Earth has evolved under the predictable pattern of solar days, i.e., exposure to relatively bright light (10,000–100,000 lux) during the day and relatively dark (0.0001–0.5 lux) during the night. During the evolution of life, organisms internalized the temporal rhythm of Earth’s rotation and eventually developed self-sustaining biological clocks. These internal daily rhythms with periods of approximately 24 h are called circadian (from the Latin, *circa* = about and *dies* = day) rhythms. The processes and structures that generate circadian rhythms are called circadian clocks. The primary circadian clock in humans is a paired cluster of about 20,000 nerve cells in the hypothalamus at the base of the brain, named the suprachiasmatic nucleus (SCN). As suggested by their name, the period of circadian clocks is approximately 24 h; daily light exposure during the day sets these periods precisely to 24 h. Having circadian clocks set to the environmental light–dark rhythms optimizes physiology and behavior and allows preparation for predictable events, such as food or mate availability, sleep times, or predator activities throughout each day.

Circadian clocks are a nearly universal feature of life on this planet [1,2], yet since the invention and wide-spread adoption of electric lights during the past 140 years, humans have managed to manipulate light duration and intensity, in both outdoor and indoor environments, so much that disrupted circadian rhythms are rampant [3]. Light exposure outside of daytime disrupts the genetic machinery underlying these endogenous circadian clocks [4,5]. As detailed below, either too much light exposure at night or too little light exposure during the day can directly disrupt central and peripheral timing mechanisms, interfere with how internal rhythms are entrained to the external environment, and dysregulate the typical and optimal 24-hour physiological and behavioral functioning of individuals. Although no direct studies in humans examine the direct effects of artificial light at night (ALAN) on the human molecular lock, converging data from human correlative studies and a plethora of data from nocturnal and diurnal animal studies indicate that circadian rhythms are disrupted, leading to similarly poor outcomes in both nocturnal and diurnal mammals, indicating that the disruptive effects of ALAN can be uncoupled from altered rest activity cycles.

A second consequence of electric lighting has been the development of around the clock human activities. These activities can directly and indirectly affect circadian clock function and include night shift work, extended hours of recreation, voluntary shifts in bed and awakening times during weekends versus the work/school week (social jet lag; see below). For example, typical night shift workers are awake, active, and eating during the daily rest phase (i.e., the night). Night shift workers especially, but anyone extending light exposure outside of the daytime hours, typically shift the timing of food intake, and often change the nutritional value of their diet [5], which also affects circadian clocks and deranges metabolism. Night shift workers are prone to cardiometabolic disorders compared to their day shift counterparts [6]. Other forms of disruption to circadian rhythms, including transmeridian jet travel across several time zones, can provoke jet lag [7,8] and also disrupt circadian function [9]. However, relatively few individuals are exposed to chronic jet lag compared to the numbers of people exposed to night-shift work, mis-timed food intake, and light at night.

Obviously, individuals cannot perform all physiological and behavioral functions all the time. Respiration [10], blood pressure [11], and heart pulse rate [12] vary throughout the day in response to activity and clock-programmed signals. Notably, energetic requirements are relatively continuous, whereas energy production or consumption is somewhat sporadic. All organisms partition temporal energetic activities across the day. Indeed, temporal partitioning of photosynthesis, metabolism, gene expression, reproduction, defense, growth, activity, and inactivity is universal among plants and animals. Disrupted circadian rhythms impair function and can increase risk for, or may provoke, diseases [13,14,15]. Inadequate exposure to light during daytime hours, and exposure to bright electric lights during the night is associated with many health disorders [16,17,18]. Indeed, most of our time is currently spent indoors [19] under light levels during the day that are typically 100 times dimmer than natural daylight, and ~1000 times brighter than natural light intensities at night. We focus this review on the role of disrupted circadian rhythms on metabolic function.

## 2. Circadian Rhythms and the Molecular Clock

Nearly every organism on Earth is the result of evolution under fairly consistent, ~24 h cycles of alternating bright, sun-lit days and dark nights as a result of the Earth’s rotation on its axis [20]. These light–dark cycles, accompanied by temperature and climate fluctuations, provide predictable environmental patterns to which organisms have adapted. This internalization has permitted them to optimize energy intake and expenditure and be prepared for environmental changes. Optimal physiological, behavioral, and reproductive timing of these events improves fitness. Given this putative biological advantage [21], circadian rhythms are seen in virtually every species to provide a predictable temporal pattern in physiology and behavior. The molecular result of this internalization, the circadian molecular clock located in the SCN, is responsible for orchestrating optimally-timed biological events, by synchronizing endogenous physiology to exogenous cues, or *zeitgebers*, ensuring internal homeostasis. The most potent cue is light [22,23,24,25], which conveys temporal information to brain centers, which then relay this temporal information via neurotransmitter and hormone signals, to all cells and organ systems.

**Figure 1:**
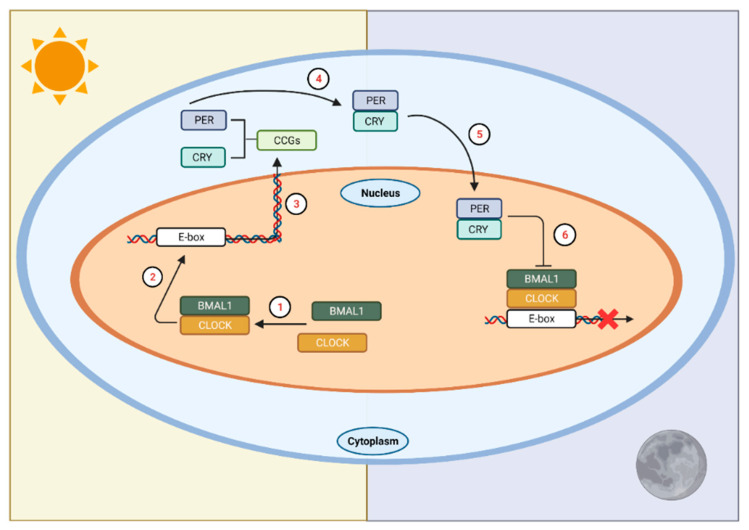
The circadian clock in mammals. In SCN neurons at the start of the circadian day, BMAL1 and CLOCK form a heterodimer (1) that binds to E-box sequences in the promoters of the Cry and Per genes (2) to activate their transcription (3). This marks the beginning of the circadian day. The gene products of Per and Cry accumulate in the cytoplasm, dimerize (4), and then form a complex that translocates into the nucleus (5) to interact with CLOCK and BMAL1, ultimately repressing their own transcription (6). This process takes approximately 24 h. LAN affects the timing of this transcription/translation cycle leading to temporal misalignments affecting physiology and behavior. CCGs = circadian clock genes. Figure was created using Biorender.com (accessed on 13 January 2022).

At the molecular level, a signaling cascade elicited by photic stimulation promotes the transcription of core clock proteins, which then initiates a series of interlocking, autoregulatory, transcriptional-translational feedback loops (TTFL) [26,27]. At the beginning of this cycle (during the biological morning), the core clock proteins circadian locomotor outputs kaput (CLOCK) and brain and muscle aryl hydrocarbon receptor nuclear translocator-like protein 1 (BMAL1) heterodimerize and translocate to the nucleus (Figure 1). The heterodimer binds to E-box sequences of their targets, cryptochrome (*Cry*) and period (*Per*), and transcription ensues. Upon accumulation of these gene products, they dimerize in the cytoplasm and eventually translocate back into the nucleus, where the cycle began. In the nucleus, they inhibit their own transcription by inhibiting transactivation of E-boxes by CLOCK and BMAL1. This process ultimately leads to the degradation and depletion of PER and CRY proteins, which disinhibits *Per* and *Cry* transcription, and the ~24 h cycle starts again.

In order to maintain the strength of this biological rhythm, this TTFL is tightly regulated at multiple levels. Specifically, regulation has been documented at the transcription factor binding and histone modifications [28], at the chromosome organization [29,30], and at the post-transcriptional [31] and post-translational [32] modification levels (see [26] for a detailed review). Auxiliary interlocked loops also exist and aid in the regulation of Bmal1 and Clock transcription. These auxiliary loops function to strengthen the rhythmicity of the primary loops in two ways. First, they help entrain the rhythms to a narrow and specific temporal range [33]. Second, they provide redundancy to the system, presumably in case of genetic mutations or other loss of function changes [34,35]. Interestingly, this ‘failsafe’ only functions as such if the co-functional gene pairs (Per1/Cry1 or Per2/Cry2) are both lacking or deficient [35]; otherwise, the system develops arrhythmicity and is unable to entrain under constant conditions. Together, these data provide evidence of the highly precise nature and critically important role of the clock genes in the regulation of physiology.

### 2.1. Phototransduction to the SCN

In mammals, rhythms are generated and maintained by the bilateral nuclei of the SCN in the anterior hypothalamus. Here, a highly specialized network of cells work in concert to maintain these rhythms in the absence of external *zeitgebers*. However, to maintain precise 24 h rhythms, these cells must continually receive input from the environment to accurately time signaling and downstream effects [36]. Although other cues, such as food and locomotor activity [37,38,39] exist, the most potent cue is exposure to sunlight early in the biological day. Photic information enters the retina [40] and travels through the monosynaptic glutamatergic retinohypothalamic tract [41,42,43] via intrinsically photosensitive retinal ganglion cells (ipRGCs) [44,45]. The IpRGCs express melanopsin, a photopigment preferentially activated by short wavelength light (~460–480 nm; appears blue), and is critical in the process of circadian entrainment. Exposure to light of this wavelength resets the molecular clock and inhibits melatonin release [46,47,48,49,50,51], an endocrine marker of time for many organisms. Notably, deletion of melanopsin in mice impedes circadian phase shifting when light-pulsed under constant dark conditions [52,53,54], whereas complete ablation of the ipRGCs themselves produces deficits in both pupillary light reflex and circadian entrainment [55].

An additional pathway that transmits both photic and nonphotic (e.g., time of food intake), information to the SCN is the geniculohypothalamic tract (GHT) [56]. The GHT primarily signals via neuropeptide Y (NPY), which serves to phase shift circadian rhythms in vitro [57] and in vivo [58], and modulates photoperiod responsiveness [59]. The phase-shifting-inhibiting action of NPY is only observed in vivo during the late subjective night [60]. Moreover, NPY serves a sleep-promoting neuropeptide by modulating noradrenergic signaling [61], and inhibiting orexin signaling [62], which modulates alertness.

### 2.2. SCN Signaling

The primary clock in the SCN communicates within the brain and periphery via neural and humoral signaling to synchronize the multiple cellular oscillators throughout the organism. Although individual clocks have the ability to oscillate independently of the SCN, it is unique because it is the only mammalian biological clock that directly receives environmental photic information. Thus, it is positioned to synchronize internal timing with that of the environment. Broadly, by providing specific timing signals (neural or humoral), the SCN couples individual oscillators within an organ or system to each other, ensuring synchronicity within the system, and setting their phase relative to the environmental time. Both neural and humoral signaling are necessary for sustaining endocrine rhythms.

Despite its widespread effects on physiological and organismal function, the SCN interfaces directly with few regions, of which its primary connections remain within the hypothalamus. Upon photic activation, the mammalian SCN communicates with the subparaventricular zone (SPZ), and the dorsomedial hypothalamic nuclei (DMH), which then relay and amplify the cellular message to downstream areas. The dorsal SPZ projects to the medial preoptic area that regulates rhythms in body temperature [63,64,65], while the ventral SPZ projects to the DMH [66]. The SCN also sends direct GABAergic projections to the DMH [66], which promotes the release of hormonal and neurotransmitter factors to regions controlling corticosteroid release, arousal [67,68] and sleep [69,70,71,72]. Thus, the SCN sends both direct and indirect input to the DMH, underlining the relevance of the strict regulation of signaling to this region and the subsequent molecular relay to regions governing homeostatic control within the organism.

## 3. Hypothalamic Control of Circadian Homeostasis and Hormone Regulation

Biological function is organized in a rhythmic, often circadian, manner, and many key life-sustaining functions, such as regulation of body temperature, metabolism, food intake and sleep are controlled by the hypothalamus. The hypothalamus is one of the central brain regions involved in coordinating organ-to-organ system communication to maintain internal homeostasis. This coordination between the nervous and endocrine systems occurs through hormonal secretion. Physiological homeostasis is regulated from autonomic, somatic, and endocrine control through complexly and tightly regulated hormone production and communication across brain regions, including energy intake, and expenditure, thermoregulation, sleep, and hormone regulation [73]. Hypothalamic hormone release functions through a cascade system induced by input received from higher brain centers responding to environmental information. These hormones travel through the hypothalamic-hypophyseal portal system to the pituitary to produce or inhibit hormones that are then transported throughout the body to interact with target organs [73]. As discussed in subsequent sections, dysregulation of the circadian system, via any of various environmental circadian rhythm disruptors (i.e., exposure to artificial light at night, night shift work, jet lag/social jet lag, or mistimed eating; see Section 4), can severely impact basic homeostatic functions. In the next sections we will briefly discuss how the hypothalamus regulates food intake and sleep, as key examples of basic homeostatic functions that are regulated in a circadian manner. We will then discuss how the SCN mediates the release of hormones that contribute to the regulation of daily rest-activity states.

### 3.1. Food Intake

Food intake is another biological process important in maintaining a healthy metabolism, from nutrient intake to optimal energy expenditure. The hypothalamus regulates appetitive behavior, food intake, and energy expenditure through hormonal release and afferent autonomic nerves [74]. The ventromedial hypothalamus (VMH) is primarily involved in satiety by receiving inputs from hormones in the bloodstream and arcuate nucleus (ARC), along with neurotransmitters, neuropeptide Y (NPY), Agouti-related peptide (AgRP), and pro-opiomelanocotin (POMC) [75]. Leptin is a hormone involved in satiety, produced in adipose tissue, and its dysregulation or dysfunction may lead to overeating. These hormones function as signals to the brain as the proportional value of fat storage relative to blood secretion levels [76]. Other hormones directly involved in food intake, include ghrelin, which directly induces food consumption through stimulating GHS-R1a in the VMH, and through an indirect orexigenic pathway from AgRP and NPY from the ARC inhibiting anorexigenic signals [77], and hypocretins/orexins, that mediate energy storage after food intake [78].

### 3.2. Sleep

Sleep is an integral biological process that affects several biological functions, including cognition, development, energy conservation, immune modulation, and others [79]. Sleep is characterized by altered brain wave activity, and reduced body movement and responsiveness to the environment [80]. The hypothalamus is one of the main brain regions responsible for playing a central role in sleep-wake regulation. The preoptic area of the hypothalamus functions by promoting sleep onset and maintenance by inhibiting arousal systems baseline, and contains inhibitory neuromodulator/transmitters galanin and gamma-aminobutyric acid (GABA) [81]. Sleep control systems are also implicated in bi-directional pathways with the SCN of the anterior hypothalamus, involved in entrainment and regulation of circadian rhythms. Notably, hypothalamic SCN oscillators, circadian rhythms, and sleep regulation have been recently implicated in playing a role in food intake, metabolism, hormone release, and temperature, suggesting its broad importance in homeostatic physiological regulation [82].

### 3.3. SCN-Mediated Hormonal Release and Function

The SCN mediates the timing of most circadian rhythms, including the daily release of many hormones associated with metabolism. In common with other circadian regulated processes, precise timing is critical. Thus, changes in environmental signals that influence circadian clock function can ultimately influence endocrine function in maladaptive ways. Interactions with peripheral clocks in the liver and pancreas, for example, that are entrained to meal timing are also critical for optimal metabolic function. As already discussed, intrahypothalamic signaling mediates the temporal output of the SCN. In the next sections we provide examples of key hormones under circadian clock control and provide the current understanding on how they communicate with the SCN.

#### 3.3.1. Melatonin

Perhaps the best known SCN-mediated endocrine response is pineal-melatonin production and secretion. Melatonin, sometimes referred to as a “sleep hormone”, at least in humans [83,84] and various diurnal species [85,86,87,88], promotes sleep. More accurately, it is a “dark” hormone, which signals night length (scotoperiod). Upon the onset of darkness (night), the SCN release of GABAergic inputs to the PVN are halted, and the paraventricular nucleus (PVN) sends projections to the intermediolateral cell column of the spinal cord [89]. From here, preganglionic noradrenergic (NEergic) neurons project to the superior cervical ganglion, which subsequently innervate the pineal gland. NE released from these fibers then promotes the biosynthesis of melatonin [90,91]. Melatonin release peaks near the middle of the night and decreases by morning [92]. Although the SCN regulates the timing of pineal melatonin release, melatonin also feeds back to the SCN, through melatonin receptor 1 (MT_1_) [93] and through melatonin receptor 2 (MT_2_) [94] signaling, to decrease neuronal firing or induce a circadian phase shift [95,96], respectively. In this context, melatonin signaling is sustained throughout much of the night and inhibited by light exposure [50,97,98].

Melatonin is not only a primary endocrine clock output, but also serves as a neuroendocrine synchronizer of molecular rhythms, both centrally and peripherally [99,100,101,102,103,104]. In rodents, melatonin induces rhythmic expression of *Per1, Bmal1, Clock,* and *Cry* in the pituitary [105,106]; pinealectomy of Syrian hamsters abolishes the rhythmicity of *Per1* mRNA expression in the pituitary without affecting its expression in the SCN or VMH [107]. Rat pinealectomy, however, leads to long-term (after three months) desynchrony of *Per1* and *Per2* expression, and decreased *Rev-erbα* amplitude in the SCN [108], suggesting that long-term loss of endogenous melatonin weakens circadian rhythmicity. Clinical studies support this hypothesis [109,110,111].

As a result of its feedback to the SCN, melatonin also plays a role in circadian entrainment [112]. Studies in humans and other mammals have demonstrated that exogenous melatonin can phase shift [113] and entrain the circadian clock of blind subjects [109,110,111]. The role of melatonin in promoting sleep itself, however, is a source of debate in the field, due to some of the data acquired from studies using nocturnal rodents. A core argument against the role of melatonin in sleep promotion results from the observation that, in these animals, melatonin is associated with wakefulness. Notwithstanding, some studies suggest that melatonin functions as a sleep-promoting signal [114,115], and it induces sedation and lowers core body temperature [116,117]. Indeed, a review of the literature suggests that administration of melatonin agonists may help in reducing sleep latency and promote sleep in patients with insomnia; however, additional clinical trials are necessary before approval for broad clinical use [118,119]. Additionally, melatonin receptors are broadly distributed (not unique to the CNS), so administration of exogenous melatonin may cause off-target effects and/or interact with other drugs, such as observed with nifedipine, an anti-hypertensive [120], although more recent data suggest protective effects [121,122].

In the periphery, melatonin has been reported to play a role in blood pressure regulation in experimental and clinical settings [103,120]. Its actions are receptor-specific; MT_1_ activation mediates vasoconstriction in both cerebral and peripheral arteries [123,124,125,126,127], and vasodilation via MT_2_ [128,129]. Other work in this field presents promising data regarding the role of melatonin in regulating blood pressure, in vascular protection after ischemic injury, and other vascular injuries [121,122,130,131,132]. Finally, in adipose tissue, melatonin synchronizes metabolic and hormonal function [133] by regulating *Per2, Clock* and *REV-ERBα,* of which *REV-ERBα* is a documented requirement for daily balanced metabolism of carbohydrates and lipids [134].

Various additional possible roles for melatonin have emerged. For instance, melatonin plays a role in T cell activation [135], and overall immune function [136,137,138,139,140,141,142], reviewed in [136,137]. It has been widely proposed as an antioxidant [143,144,145,146], particularly in the context of recovery from ischemia/reperfusion injury [121,143].

A more nuanced role for melatonin has been proposed for the aged. Specifically, given the association between decreased melatonin production in the early stages of Alzheimer’s disease (AD), the role of melatonin in sleep promotion (in humans), and the function of sleep in clearing the brain from metabolites and toxins, melatonin has been proposed as a promising therapeutic for those at risk [147]. Moreover, a preclinical study analyzing the relationship between sleep and the accumulation of Aβ, determined that sleep deprivation or orexin administration increases Aβ in interstitial fluid, suggesting a role for sleep and wakefulness-regulating molecular players in AD pathology [148]. Further, in the context of AD, and considering its antioxidant capacity, melatonin has also been proposed as an ‘anti-aging’ agent, as it is capable of stimulating antioxidant enzymes and re-establishing the mitochondrial membrane integrity [149]. Further pre-clinical and clinical research is required to fully understand the interconnectedness of these pathways and the molecular mechanisms that mediate their effects.

Despite the mounting literature on the systemic effects of melatonin, more work is required to fully understand (1) endogenous versus exogenous melatonin effects, (2) its central versus peripheral effects, (3) differential effects on diurnal versus nocturnal species, and (4) sex differences in response to its administration.

#### 3.3.2. Glucocorticoids

Glucocorticoids (GC) are steroid hormones produced by the adrenal cortex, and are involved in several physiological processes, such as metabolism [150], immune response [151], cardiovascular function [152,153], and reproduction [154]. As many homeostatic drivers of physiology, the production of GC is driven by circadian rhythms and is tightly regulated. Neuronal activity in the SCN stimulates the PVN to initiate the release of corticotropin-releasing hormone, which then signals to the anterior pituitary to release adrenocorticotrophin, which, in turn, communicates to the adrenal cortex to release GC. Over time, GC feed back to the hypothalamus and pituitary to block secretion of their precursors, and consequently, their production. There is no GC feedback directly to the SCN [155]; however, the arcuate nucleus serves as the “stress sensor”, providing feedback to the PVN about corticosterone levels, and thus, modulating its release [156]. As a result, GC are released at specific times of the day, and in response to specific events (e.g., corticosterone release as a result of restraint), and display cyclic decreases and increases in their circulating concentrations. Cortisol secretion, for instance, begins prior to awakening, rises in the morning, peaking around the time of awakening [157,158]; elevated GC increase glucose availability in anticipation of the increase in energy demands associated with wakefulness [159]. In rodents and other nocturnal animals, the cycle of corticosterone secretion is reversed, with the peak observed around dusk, but coinciding with the beginning of their active phase [160].

The GC play an important role in overall animal physiology, particularly in the stress response. During times of stress, the release of GC and epinephrine suppresses energy storage and shifts towards usage of adipose and liver stores [161,162]. Thus, GC released as part of the stress response may also shut down cellular processes that require ample energy resources, such as humoral immune responses, digestion, growth, and reproduction [161,163].

## 4. Changing Environment and Consequences on Hormonal Rhythms

Environmental changes such as widespread adoption of artificial light at night and the subsequent shift in human activities that affect the internal timing system cause a myriad of health disturbances, both immediately (such as lack of sleep or altered energy intake) and long-term (such as the accumulation of risk factors to cardiometabolic disorders and elevated risk of developing endocrine, gastrointestinal, and neurological disorders). Over the past few decades, the field of chronobiology has focused on some of these unintended consequences and has focused research efforts on understanding the interplay between these technological developments and their long-term effects on humans and other organisms. Because light has neuromodulatory effects on the SCN, and the SCN regulates downstream secondary oscillators, light and circadian rhythms have been central to this work.

The circadian clock has direct influence on hormonal rhythms in the endocrine system. As such, exposure to changing environmental conditions that affect circadian rhythms can have adverse physiological consequences on health, particularly affecting the hypothalamus and its function in communicating and modulating signaling between the nervous and endocrine systems. Imbalances or alterations to oscillatory hormonal rhythms can unfavorably affect cellular and molecular processes in physiology and increase risk for diabetes, metabolic disruption, and altered hormonal signaling [164]. Increasing evidence highlights the role of light pollution negatively affecting human and non-human animal health, including disruptions to molecular circadian clocks, metabolism, and hormonally-driven physiological functioning, in addition to, or in parallel with, disrupted sleep-wake cycles.

### 4.1. Artificial Light at Night

Circadian clocks rely on light as the primary *zeitgeber* responsible for synchronizing endogenous biological rhythms to the 24 h light–dark cycles. Therefore, mammals are particularly vulnerable to circadian disruption by exposure to low, or even dim, levels of artificial light at night (ALAN), below the threshold necessary to directly disrupt rest activity cycles. Exposure to ALAN is now ubiquitous. Humans undergoing hospital stays, transmeridian travel, or night shift work are exposed to constant lighting, which can disrupt circadian rhythms and clock genes resulting in misalignment of circadian-controlled physiology and overall physiological homeostasis [165,166,167]. Animal models of nocturnal light exposure have provided substantial evidence supporting the detrimental effects on cellular to behavioral physiology [166,168,169,170].

Metabolism and circadian rhythm disruption is well characterized. Time of day alters energy expenditure and disrupted circadian rhythms can increase metabolic abnormalities and elevate the risk for obesity. Chronic exposure to ALAN increases body mass and impairs glucose processing while maintaining equivalent caloric intake and locomotor activity patterns [169]. Insulin is another circulating hormone that is affected by ALAN and disrupted circadian rhythms, independent of food intake; although the precise mechanisms through which desynchronization of central and peripheral clocks contributes to insulin resistance has yet to be determined, a recent review of the literature [171] proposes two possible mediators of this interaction: the phosphatases belonging to the pleckstrin homology leucine-rich repeat protein phosphatase family and the deacetylase Sirtuin1 [172]. ALAN decreases glucose tolerance and insulin sensitivity in rodent models [168,173] and in human studies [174]. Just one night of 100 lux light exposure during sleep affects various cardiometabolic measures; specifically, ALAN increases nighttime heart rate, decreases heart rate variability, and increases next-morning insulin resistance in humans [174]. Further, other aspects of metabolism including energy expenditure regulated by brown adipose tissue (BAT), are altered with prolonged lighting duration in rodent models, by decreasing β3-adrenergic intracellular signaling/sympathetic input to BAT, and impairing fatty acid uptake from triglycerides [175]. Thermogenesis and body temperature are also affected by ALAN; nighttime light exposure increases brain and core body temperature in a rodent model [176].

ALAN has been directly implicated in altering central neuroendocrine rhythms such as the hypothalamo-pituitary-adrenal (HPA) axis, which comprises hormonal feedback mechanisms that regulate axis activity [177]. Corticotropin-releasing hormone (CRH) and arginine vasopressin (AVP) are involved in the daily release of corticosterone and are regulated by clock genes [178]. In mouse models of circadian rhythm disruption, exposure to bright or constant ALAN increases glucocorticoid concentrations [179], whereas dim ALAN (i.e., 5 lux) does not alter glucocorticoids [169]. It is possible that these null results in response to 5 lux reflect phase advanced plasma glucocorticoid concentrations after exposure to ALAN, so that low values reflect differences in biological time of day [180]. These studies suggest that the intensity of lighting plays an important role in hormonal and metabolic disruption, but species differences may also play a role in the observed effects.

Melatonin as one of the hormones predominantly involved in circadian cycles, relies on external lighting cues to regulate its production and secretion, and is affected by ALAN. In addition to melatonin’s role in nocturnal physiology, a core circadian rhythm, there are several receptor-mediated physiological involvements, including immune-modulation and free-radical scavenging [181]. In both human and rodent studies, the extent of suppression is dependent on duration, intensity, and wavelength [182]; notably, dim or constant lighting suppresses melatonin release onset, and shortens melatonin duration [183]. Circulating melatonin additionally plays a role in regulating glucose and plasma leptin rhythms [184], and suppressed melatonin due to exposure to ALAN can affect metabolic regulation. Further, disrupted melatonin concentrations have been associated with changes in thyroid, adrenal, and reproductive hormone rhythms [185,186], suggesting that the effects of ALAN and disruptions to melatonin rhythms may further exacerbate neuroendocrine and metabolic disruptions.

### 4.2. Shift Work and Jet Lag

Disruption of circadian rhythms can occur through other means such as shift work, erratic social schedules (i.e., social jet lag), and traveling across time zones (i.e., jet lag). These can impair sleep-wake cycles, and, in turn, can affect other physiological processes, resulting in a myriad of adverse health consequences [187], such as increased risk for weight gain and metabolic disruption, along with risk for diabetes, cardiovascular disease, and cancer.

Night shift work refers to jobs that encompass overnight work (e.g., 18:00 h–7:00 h), or more generally, beyond the common “9 to 5”. Presumably, because humans evolved to be active (i.e., work) during the day and inactive (i.e., sleep) during the night, their physiology also evolved to maximize metabolic efficiency during the day, and optimize recovery and removal of waste and toxins at night [188,189,190]. Indeed, data from both basic and clinical studies support the role for metabolic and hormonal dysregulation associated with night shift work [191,192]. Circadian misalignment from exposure to 8 h phase advance and/or delays, shifted glucose tolerance and altered insulin sensitivity during the light (inactive) phase in rodents [173,193]. Other manipulations that disrupt circadian rhythms, such as social jet lag, have also been implicated in dysregulating hormonal rhythms that contribute to metabolic syndrome. In rodents, shifting of light/dark cycles and/or persistence of dim ALAN can increase glucose intolerance, body mass [169], and white adipose tissue with changes to metabolic profiles in the liver [194], and social jet lag increases body mass gain by overconsumption [195].

In the context of clinical data, a study using ten 28 h “day” shift-work paradigm in humans reported dysregulated plasma leptin, insulin, glucose, and cortisol concentrations [196] and observational studies support the increased risk of obesity associated with night shift workers [197]. Other studies examining food intake in relation to social jet lag, noted that social jet lag is associated with later meal times and poor nutritional continent in meal choices [198]. Nonetheless, it is worth noting that the shift work schedule maintained, including the number of consecutive hours worked, the number of consecutive days worked in a nocturnal shift, and the pace with which the worker alternates between work and rest days, as well as how often they shift between day and night shifts, may affect health outcomes, and may differentially affect systems. Thus, the effects of night shift work may strongly depend on the type of rotating schedules [199,200,201].

### 4.3. Mistimed Food Intake

The molecular circadian clock is responsible for coupling the regulation of metabolic and cellular processes to time of day. Meal timing and food composition are additional *zeitgebers* that can entrain circadian rhythms [37] and are typically limited to the active phase of organisms. Thus, shifting meal timing as a result of social or work responsibilities (night-shift work or other social schedules), can also phase shift internal clocks to functionally alter endogenous circadian rhythms.

Unfortunately, for humans, mistimed eating usually indicates eating during the nighttime. However, because human physiology developed with sleep typically occurring at night, eating during the nighttime is inherently physiologically “incorrect”. Further, humans are adapted to dark nights, with physiology programmed to rest and recover during that time; thus, night shift workers’ exposure to artificial light also inherently affects the molecular framework of circadian rhythms, which ultimately impairs downstream physiology. Thus, disentangling the contributions of these individual cycle disruptors (nocturnal light exposure, lack of nocturnal sleep, etc.) from that of mistimed daily eating, becomes very complex. Nonetheless, regardless of the mechanism of how misalignment happens, data demonstrate that eating during the nighttime impairs circadian function [202,203], and overall decreases the nutritional value of the diet [198,204], increasing fat, saturated fats, and cholesterol consumption and total caloric value.

Studies using nocturnal rodents have provided some insights on the effects of light on feeding behavior independent of sleep. Studies using low-level ALAN in mice have determined that exposure to ALAN shifts the time of food consumption [169] to that of the biological inactive phase, without affecting sleep quality [205]. However, a more recent study using nocturnal rats reported that exposure to ALAN alters the amplitude of the rhythms of REM and non-REM sleep [206]. Nonetheless, many dysfunctional metabolic parameters arise, such as impaired glucose utilization [169,173,207,208] and insulin sensitivity [173]; however, when the timing of food intake is restricted to the active phase, some of the metabolic disorders are reversed [173,209]. Moreover, studies with diurnal rodents have also demonstrated misaligned rhythms and disturbed metabolic function when exposed to ALAN and/or shift work models [173,210]. Thus, it is possible that the metabolic effects of misaligned rhythms via exposure to ALAN are independent of sleep itself, especially when we consider that SCN neurons are most active during the daytime, irrespective of whether the organism is diurnal or nocturnal [211]. Indeed, the study [206], in which it was reported that ALAN impairs sleep in rats, also reported changes in SCN clock gene expression, without the typically reported changes in body mass and glucose tolerance observed in mice. Thus, the mechanisms mediating the effects of ALAN on sleep and metabolism may not be coupled or may differ by species. A recent review of the “light-at-night” literature in the context of mammals, provides evidence to suggest more nuanced effects on sleep in diurnal versus nocturnal creatures [212]. In sum, this body of work supports the notion that circadian disruptors can differentially affect individual circadian processes, and that the interaction of individually affected pathways and processes may result in compounding and complex effects that need to be further examined.

## 5. Changing Environments, Misaligned Circadian Rhythms, and Resulting Disorders

Circadian rhythms allow organisms to synchronize their physiology and behavior with cues from the external environment to maximize resources and reduce energy expenditure. Again, these rhythms have evolved to be aligned with the 24 h solar day. This provides a relatively simple, but powerful, cue that entrains physiology to efficiently use energetic resources when the body is optimized to do so (organism’s active phase), and effectively metabolize energy stores to maintain stable glucose supply during fasting (organism’s inactive phase). Thus, any stimulus that impairs the receipt of the temporal cue provided by light and the circadian timing system, threatens physiological homeostasis.

Non-natural environmental changes, as the ones described in the prior section, have a direct effect on various aspects of behavior, metabolism and overall physiological homeostasis. Next, we provide a brief overview of the relationship between circadian rhythms and endocrine, metabolic and cardiovascular disorders.

### Endocrine, Metabolic, and Cardiovascular Diseases

The human metabolic and cardiovascular systems are especially vulnerable to nocturnal rhythm disruption, as during these hours, the body is not prepared for the energetic demands of activity and the processing of nutrients. As described, the rhythm of energy intake and expenditure is carefully regulated to coincide with the release and regulation of hormones that allow the body to signal the need for energy, (i.e., hunger, ghrelin), and enzymes that contribute to the extraction of nutrients (e.g., amylase, lipase, etc.), with that of the hormones that signal satiety (i.e., leptin) and promote digestion. Night shift workers, however, threaten the internal metabolic balance as they displace their sleep-wake and food consumption schedules to biologically incompatible times. For instance, consumption of a large meal at night uncouples the relationship between plasma glucose and insulin concentrations, which can lead to metabolic dysfunction [213]. Indeed, severing circadian and behavioral rhythms under a chronic night shift work paradigm, uncouples synchrony between hormones that follow an ~24 h cycle (endogenous rhythm, i.e., glucose, epinephrine, and cortisol), and those that shift as a result of behavior (i.e., leptin, insulin, and norepinephrine). Leptin production decreases, with an increase in glucose concentration, despite concurrent increased insulin production [196]. Decreased leptin increases hunger, which increases food intake, with a consequent decrease in energy expenditure. Together, these contribute to the increased plasma glucose concentrations and an obese phenotype. Moreover, under this chronic shift work paradigm, the cortisol peak occurs during the end of the wake period/beginning of sleep, which contributes to insulin resistance [214,215] and hyperglycemia [216,217].

In the context of night shift work, or otherwise shifted (human) behavioral schedules (i.e., being active/awake at night), biological routines such as food consumption are also altered. As evidenced from both preclinical and clinical data, glucose metabolism is also influenced by mistimed eating. For instance, night eating syndrome is associated with obesity [218], and in overweight female night shift workers, compared to overweight day shift workers (with no difference in BMI), the post-meal suppression of ghrelin production was blunted, resulting in elevated waist circumference, greater energy intake, impaired sleep, insulin insensitivity, increased triglycerides and increased C-reactive protein (CRP) [219]. These risk factors have all been associated with the development of type 2 diabetes [220,221,222,223] and pathological cardiovascular events (cardiovascular diseases; CVD) [224,225,226,227]. The CRP, an inflammatory biomarker that is present in most cardiac pathologies, and whose production may be triggered by metabolic hallmarks of diabetes—high glucose, adipokines, lipoproteins and free fatty acids—plays a role in the development and progression of most CVD, with immunomodulatory roles in the humoral innate immune and complement responses, as well as in vascular function [201]. Although it remains unspecified whether CRP acts as a regulator or amplifier of the immune response. Regardless, detection of CRP during risk assessment of CVD, namely, myocardial infarction, greatly improves its prognostic power [228], and can help prevent another event within the subsequent 10 years [229].

In the context of vascular function, CRP inhibits endothelial nitric oxide synthase (eNOS) production [230], which is necessary for the synthesis of nitric oxide (NO), the primary endogenous vasodilator. Thus, decreased production can lead to dysregulated vasoconstriction, leukocyte adherence, platelet activation, oxidation, impaired or uncontrolled coagulation, and vascular inflammation [231]. However, under exacerbated inflammatory states, also mediated by CRP, NO production increases, and along with other reactive oxygen species, can lead to oxidative stress, which in turn, promotes vascular pathologies such as atherosclerosis [232]. In the context of shifted sleep schedules or sleep deprivation, which also have detrimental effects on metabolic parameters, (e.g., exacerbated insulin resistance, [214], these data suggest possible overlapping mechanisms for the development and persistence of cardiometabolic disorders such as obesity [197,233,234,235], diabetes [6,236,237], hypertension [238,239], and other CVD. Further, a meta-analysis directly assessing the relationship between shift work and the development of CVD, determined that shift work increases the risk of myocardial infarction, cerebrovascular accidents and coronary disease [240], and increases the risk of reperfusion injury following myocardial infarction [241], lending evidence to support a relationship between disrupted circadian rhythms and the development of cardiovascular pathologies.

Lastly, lifestyle choices may also interact with disrupted rhythms to exacerbate metabolic pathology. Studies have documented shift workers’ elevated risks for overall smoking [242], and are also more prone to smoke during their shifts, in order to stay awake, curb hunger and/or relieve stress [242,243,244,245]. Similarly, alcohol overconsumption has been documented in night or rotating shift nurses, potentially as a sleep aid [246], with decreased activity (exercise) [247,248]. These behaviors, together with pre-existing risk factors, such as elevated triglycerides [249,250], uncontrolled hypertension [236,239], and/or dyslipidemia [251], compounded by the physiological dysfunctions resulting from circadian disruption through shift work, may interact to promote a prediabetic state [233].

Taken together, these converging lines of research provide ample support for the relationship of disrupted circadian rhythms and cardiometabolic dysfunction. However, the relationship, although seemingly direct (i.e., exacerbated metabolic function co-occurs with disrupted circadian rhythms) may not be linear. Multiple, otherwise innocuous, daily routines, such as food intake and sleep, may occur when other physiological processes are endogenously-timed to occur, thus impairing their biological function and homeostasis. Moreover, coupling those physiological aspects to behavioral nuances (exercise or lack thereof, dietary habits, disordered substance use, etc.), may further complicate the interrelations of all the above.

## 6. Strategies to Remediate Effects on Disrupted Rhythms in Humans

### 6.1. Dark Nights

Various strategies to mitigate the effects of our changing environment and their noxious effects on circadian rhythmicity can be taken, and may involve only slight modifications of behavior, at a low cost. During the night, exposure to light in the short wavelength (“blue light”) can phase-shift the body’s internal rhythms and delay melatonin release. Thus, limiting exposure to blue light or filtering it out can help retain synchronized rhythms. For instance, if the need arises to work late into the night and/or be exposed to blue-light-emitting devices, then one may employ the use of glasses that filter this wavelength light [252], and limit their disruptive effects [253]. These glasses function to reduce the component of light that stimulates the intrinsically photosensitive retinal ganglion cells, and thus, improving the dim-light melatonin onset. Their use has been demonstrated to improve sleep and mood [254]. In the bedroom, using blackout curtains may also help.

### 6.2. Decrease Blue Light Exposure in the Evening

A related strategy consists of implementing a “naturalistic light environment” approach. This strategy requires modifying environmental lighting (with the use of a specialized light system) to become “circadian lighting”, where daily illumination dynamically changes throughout the day to reflect the external day conditions. Specifically, under this paradigm, light is depleted of blue wavelength-rich light during the evening and night hours [255]. Using a cross-over design decreased suppression of melatonin levels, a dim-light melatonin onset phase-advance, and an increase in total sleep were observed in the blue-depleted light conditions, suggesting that modulating nocturnal artificial lighting to emulate that of the external environment, could be a promising strategy. However, this experimental protocol used only five days in each experimental arm, so longer-term experiments should be conducted to elucidate the sustained effects of this manipulation.

### 6.3. Early Morning Bright Light

Blue light, however, is not all “bad”. Exposure to bright light (bright light therapy; BLT) [256,257,258] early in the biological morning [259] can also help synchronize individuals’ rhythms in a variety of contexts [256,260,261,262]. Indeed, BLT has been used to treat the symptoms of seasonal affective disorder (SAD) when administered early in the biological day [256,257]. BLT has also been proven effective as a second line of treatment for non-seasonal depression [261], and has been used to treat symptoms such as lethargy, decreased alertness, and blunted mood in patients with dementia [260]. BLT has also been effective in resynchronizing rhythms in patients diagnosed with ADHD, an evening chronotype and sleep problems [263,264].

### 6.4. Food Restriction to the Active Phase

Another strategy to synchronize endogenous rhythms is limiting food intake to the active phase. As discussed, metabolism is tightly regulated by the circadian clock [134,168,202,265], and energetic cues that alter the internal balance threaten physiological homeostasis, leading to increased risk for obesity, glucose and insulin imbalance, and cardiovascular disorders [204,266,267,268,269,270]. Food restriction has been demonstrated to re-entrain rhythms and limit weight gain in rodents [169,271] and humans [37,272]. Moreover, a recent study demonstrated that caloric restriction in addition to circadian alignment of feeding further optimized the beneficial effects of timed-feeding in mice, viz., weight management and, importantly, extended the animal’s lifespan and delayed age-related gene expression changes in immune function and metabolism [273].

### 6.5. Melatonin Supplementation

Oral melatonin is commercially available in the US, marketed as a dietary supplement, specifically, as a sleep aid, which has been reported to reduce sleep latency onset and increase sleep duration [84]. However, because of its status as a dietary supplement, it is not regulated by the US Food and Drug Administration; thus, true melatonin content is not certified. A 2017 study analyzing the melatonin content of 31 commercially available supplements determined that melatonin greatly varied across supplements from 83–478% of the advertised melatonin content [274,275]. Further, 26% of the supplements contained serotonin, which can be a health risk. This, perhaps, is not surprising because the use of melatonin in the US has become commonplace as a relatively inexpensive and easily-accessible supplement. Indeed, the use of melatonin supplements has quintupled between 1999 and 2018 [276,277]. Overall, the documented increased use of melatonin in the US raises concerns of its safety as a result of the lack of regulation, especially in the context of vulnerable populations, such as children or the elderly. It is worth highlighting that the US is one of few countries that makes melatonin supplements accessible over the counter. In light of this information, the use of melatonin should probably only be used under the direct supervision of a physician and sourced from a vetted supplier.

## 7. Recommendations for Night Shift Workers and Future Work

It is unlikely that night shift work will disappear, yet it is very likely that many of the long-term effects of disrupted circadian rhythms on human function remain to be uncovered. The strategies that we provided to potentially remediate the effects of circadian rhythms on humans have been, thus far, demonstrated to be helpful in mitigating some of the effects of altered nocturnal rhythms, at least in the short-term and for non-chronic rhythm disruption. Their success in mitigating long-term pathological effects on night shift workers remains to be determined.

Additional strategies are currently recommended for night shift workers; these are primarily aimed at adjusting individuals’ daily environmental conditions in a manner that facilitates an appropriate circadian phase shift, and aligns their internal rhythms to their shifted 24 h light–dark schedule. For instance, recommendations include keeping a consistent schedule for at least 3+ days (not continually alternating between day and night shifts), and keeping shifts under 11 h. Other recommendations include not going to bed immediately after the night shift ends, but rather going to bed later in the day and waking up a few hours before the next night shift, in common with what most diurnal workers do. When it comes to preparation for sleep, it is recommended that one avoids caffeine 3–4 h before going to bed; consuming melatonin supplements 1–2 h before bedtime, and keeping the sleeping environment cool, with minimal exposure to light sources among others [278,279]. The use of sleep masks and black-out curtains in the sleeping rooms is also recommended. Together, the goal is to mimic the conditions one may encounter during the natural active/inactive phases (for instance, the nocturnal decrease in body temperature). Nonetheless, further longitudinal data need to be collected and analyzed to determine best practices and to develop standard operating procedures for widespread use in the night shift work industry. These studies should focus on strategies to best adapt to and properly function within the chosen 24 h rest-activity cycle, as well as investigate the long-term effects of such lifestyle, on metabolism and overall health.

## 8. Conclusions

Metabolism is a key homeostatic function that is integral to organismal functioning and regulated in a circadian-dependent manner. Any alteration that threatens metabolic temporal equilibrium could have potentially negative, long-term, and persistent repercussions. Here, we highlight the relationship between hormonal metabolism and misaligned rhythms as a result of exposure to light at night, night shift work, jet lag, and mistimed eating. Substantial data have been reviewed from preclinical and clinical settings that reveal interconnected relationships among factors that correlate with impaired rhythmic physiological function (e.g., night shift work), pre-existing conditions (e.g., obesity), and lifestyle choices (e.g., smoking) to exacerbate pathological states. Nonetheless, more research is required to further understand these phenomena, and how they differentially affect individuals by age and sex to develop strategies to mitigate the negative effects and develop appropriate policies to protect those in positions where environmental conditions are misaligned with endogenous physiology (e.g., night shift workers).

Various strategies to counter some of the sequelae of disrupted internal rhythms are available and promising, but further research into their effectiveness, long-term applicability, and the differential effects by age, sex, and possibly, chronotype (individuals’ natural inclination in reference to time of day when sleep is preferred or most alert) remain unspecified. Moreover, regulatory measures should be considered to ensure that both pharmacological and non-pharmacological treatments are as advertised (e.g., precise drug content and functional blue light filters). Given the increasing need to extend daily activity well into the nighttime for work, study, and social activities, awareness of preventative measures should be widely-disseminated for human health and safety.
